# Unveiling the nongenomic actions of thyroid hormones in adult mammalian brain: The legacy of Mary B. Dratman

**DOI:** 10.3389/fendo.2023.1240265

**Published:** 2023-09-29

**Authors:** Nilkanta Chakrabarti, Pradip K. Sarkar, Arun K. Ray, Joseph V. Martin

**Affiliations:** ^1^ Department of Physiology, University of Calcutta, Kolkata, West Bengal, India; ^2^ (CPEPA-UGC) Centre for Electro-Physiological and Neuroimaging studies including Mathematical Modelling, University of Calcutta, Kolkata, West Bengal, India; ^3^ Department of Basic Sciences, Parker University, Dallas, TX, United States; ^4^ Department of Molecular Medicine, Bose Institute, P-1/12 CIT Scheme VII-M, Kolkata, India; ^5^ Biology Department, Center for Computational and Integrative Biology, Rutgers University, Camden, NJ, United States

**Keywords:** triiodothyronine, thyroxine, thyronine, thyronamine, synaptosomes

## Abstract

A comprehensive review was conducted to compile the contributions of Mary B. Dratman and studies by other researchers in the field of nongenomic actions of thyroid hormones in adult mammalian brain. Dratman and her collaborators authored roughly half of the papers in this area. It has been almost fifty years since Dratman introduced the novel concept of thyroid hormones as neurotransmitters for the first time. The characterization of unique brain-region specific accumulation of thyroid hormones within the nerve terminals in adult mammals was a remarkable contribution by Dratman. It suggested a neurotransmitter- or neuromodulator-like role of thyroid hormone and/or its derivative, 3-iodothyronamine within adrenergic systems in adult mammalian brain. Several studies by other researchers using synaptosomes as a model system, have contributed to the concept of direct nongenomic actions of thyroid hormones at synaptic regions by establishing that thyroid hormones or their derivatives can bind to synaptosomal membranes, alter membrane functions including enzymatic activities and ion transport, elicit Ca^2+^/NO-dependent signaling pathways and induce substrate-protein phosphorylation. Such findings can help to explain the physiological and pathophysiological roles of thyroid hormone in psychobehavioral control in adult mammalian brain. However, the exact mode of nongenomic actions of thyroid hormones at nerve terminals in adult mammalian brain awaits further study.

## 1 Introduction

Three centuries after the initial anatomical description of the thyroid gland (1), L-triiodothyronine (T3) was recognized as the active form of thyroid hormones (THs) (2). Subsequent studies have continued to unveil the functional roles of TH. The identification of nuclear TH receptors (nTR), and the elucidation of their transcriptional properties (3) unlocked new vistas for TH research in most tissues. The cloning of nTR and its isoforms (4) further strengthened the knowledge of the molecular foundations of TH endocrinology in peripheral tissues. However, little evidence of TH-induced transcriptional activity was detected in adult mammalian brain (5). Hence, the mature mammalian central nervous system (CNS) was identified as an unresponsive tissue to TH. Meanwhile, researchers acknowledged the relationship between adult-onset thyroid dysfunction and various neurological and psychological anomalies in adult humans. Two major isoforms of nTR (nTRα, nTRβ) were demonstrated in adult mammalian brain. Still, the transcriptional mechanism of TH action could not explain the behavioral sensitivity to TH in the adult mammalian brain (6).

A parallel group of researchers including Dr. Mary B. Dratman, turned their thoughts in a different direction. THs are synthesized from the amino acid tyrosine, the known precursor to catecholamines. The final synthesis of catecholamines occurs by decarboxylation and other modification reactions (7). The structural chemistry implied that T4 and T3 also could be decarboxylated in a similar way, resulting in tetraiodothyronamine (T4AM) or 3,3’,5’-triiodothyronamine (T3AM) and derivatives. These amines might exert aminergic actions like classical catecholamines. The hypothesis that aminergic TH derivatives could have neurotransmitter-like functions was proposed (6, 8). To examine this hypothesis, the differential localization of THs within the mammalian CNS was examined. Distinct brain regional distributions of radioactivity were observed following administration of radiolabeled THs (9). The finding of radiolabeled THs in synaptosomes, a nerve ending preparation without cell nuclei, indicated a potential action at the synapse. The concept gradually evolved for a role of TH derivatives in having neurotransmitter-like actions. This idea suggested that TH derivatives might have nongenomic effects in brain, like other neurotransmitters do. Although genomic pathways for TH action in adult brain are now known (10–12), Dratman’s research was fundamental in identifying the predominantly noncanonical actions of THs in mature mammalian CNS (13–15).

The objective of the present paper is to perform a comprehensive review of the research contribution by Dratman and studies by other researchers to explore nongenomic mechanism of action of THs in adult mammalian brain.

## 2 Methods of comprehensive review

We have executed a comprehensive review to find literature related to the nongenomic actions of TH at nerve terminals in adult mammalian brain. The literature search was conducted using the following key words: ‘adult’, ‘mammalian’, ‘brain’, ‘synapse’, ‘nerve terminal’, ‘synaptosome’, ‘thyroid hormone’, ‘T3’, ‘T4’, ‘triiodothyronine’, and ‘tetraiodothyronine’ with different combinations, using a ‘Pubmed’ search. Subsequent manual curation of the articles included criteria such as articles in ‘English language’ and articles focusing only on synaptic regions of adult brain.

**Figure 1:**
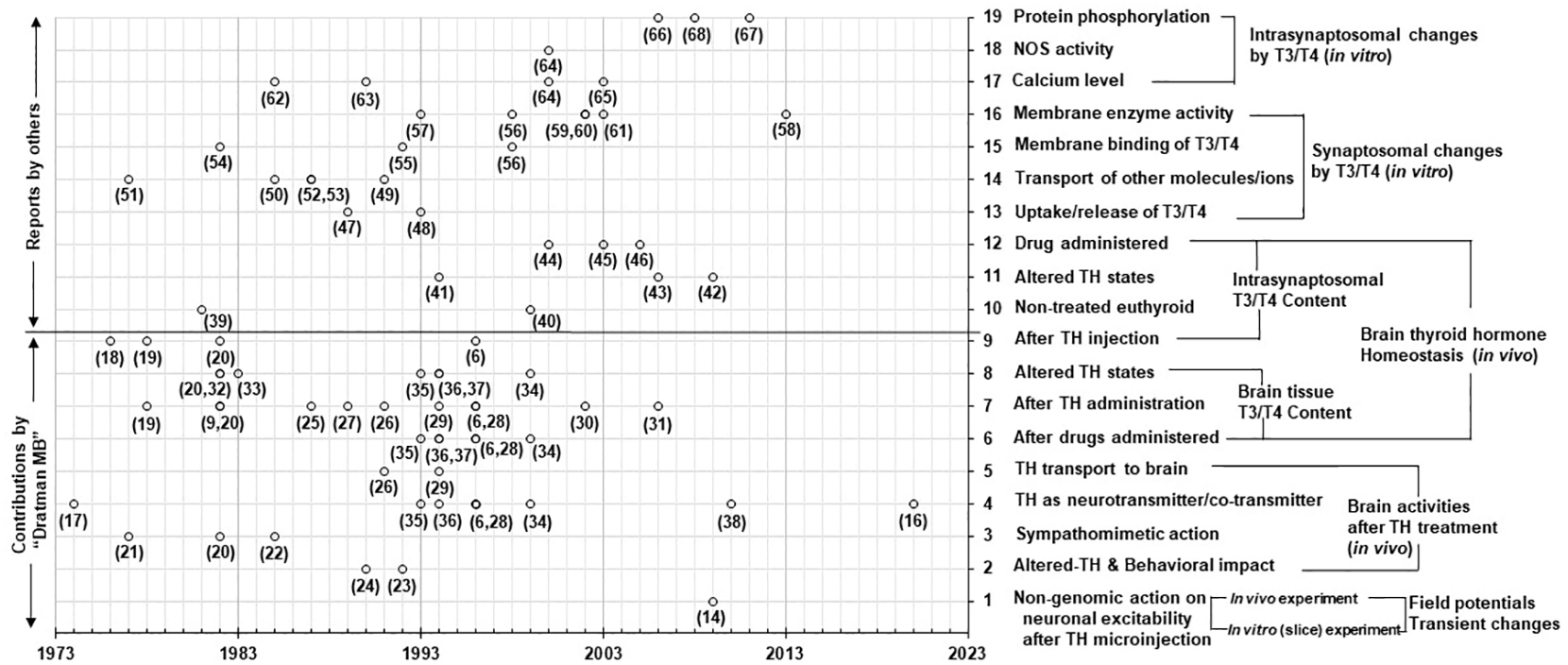
A chronological view of literature addressing the conceptual development of the nongenomic action of TH in adult mammalian brain. The graph represents the year-wise (X-axis) publications of the research articles (hollow dots) with development of the concepts (Y-axis, right-hand). The numbers close to the dots correspond to the reference numbers listed in the review. All publications have been curated through the comprehensive review of literature in Pubmed searches. The text (Y-axis, right-hand) represents the categories (19 levels) of experimental results found in the publications. For example, the experimental results at level-9 (Y-axis; right-hand) indicate the publications in support of the concept originated and developed by ‘Dratman MB’ concerning T3/T4 levels in synaptosomes isolated from rat brain after radiolabeled TH injection to the animals. The contributions of other findings of ‘Dratman MB’ in support of various concepts are as mentioned in level-1 to level-8 (Y-axis, right-hand). The research publications by others relate only to synaptosomal studies for both *in vivo* and *in vitro* experiments as mentioned from level-10 to level-19 (Y-axis, right-hand). Some research articles include more than one level of concept.

A similar search was conducted using above-mentioned keywords with ‘Dratman MB’ as an additional keyword. Out of a total 345 articles (including duplicates), 58 articles were selected for the present study. Of these, 26 articles were published by Dratman et al. Notably, 32 articles (other than publications of Dratman et al.) were found to be related to TH action on synaptosomes. The selected 58 articles were categorized based on the topics of interest. The chronological development of the concepts by Dratman et al. and separate studies by other researchers is presented graphically in **Figure 1**.

## 3 Nongenomic actions of TH in adult mammalian brain

A major contribution of Dratman and her collaborators is the notion that THs have actions in mature mammalian CNS by noncanonical mechanisms. The effects of THs to bind to nuclear receptors, which in turn interact with DNA to regulate gene expression, are not as prominent in mature mammalian brain as in developing brain (3, 5, 6).

### 3.1 Summary of findings of the comprehensive review

In the comprehensive review, we have focused on the details of the contributions of (a) ‘Dratman et al. (levels 1 to 9 on the right-hand Y-axis) and (b) separate studies by other investigators (levels 10 to 19 on the right-hand Y-axis) in the field of nongenomic action TH in adult mammalian brain (see **Figure 1**).

The research publications by Dratman were categorized into nine different types of findings (levels 1-9 on the right-hand Y-axis);. Her publications included concepts of the brain TH homeostatic mechanism (i.e., the maintenance of the T3/T4 levels in nerve terminals in level 9 and brain tissues (levels 6-8) under different experimental conditions). Additional Dratman publications (level 5) included the transport of TH to brain, the role of T3 and/or its derivative 3-iodothyroamine (3-T1AM) within adrenergic systems, the sympathomimetic actions of TH, the behavioral impact of TH and the direct nongenomic action of T3 on neuronal activation. Notably, all contributions of Dratman et al. include research outputs through *in vivo* experiments with findings of T3/T4 and their metabolites in brain tissues, nerve terminals and isolated synaptosomes.

The research publications by other workers under levels 10-19 (**Figure 1**; right-hand Y-axis) include both *in vivo* (levels 10-12) and *in vitro* experiments to find the thyroid status and effects of T3/T4 on synaptosomes isolated from adult rodent brain.

The research publications concerning drug administration to rodents included blockers of synaptosomal norepinephrine (NE) transport (level 12) or neurotoxins for the locus coeruleus (origin of adrenergic nerve cells in brain) described by Dratman et al. (level 6). Additional studies, by other researchers, employed antidepressant drugs (level 12).

#### 3.1.1 Contributions of Dratman

**Figure 2:**
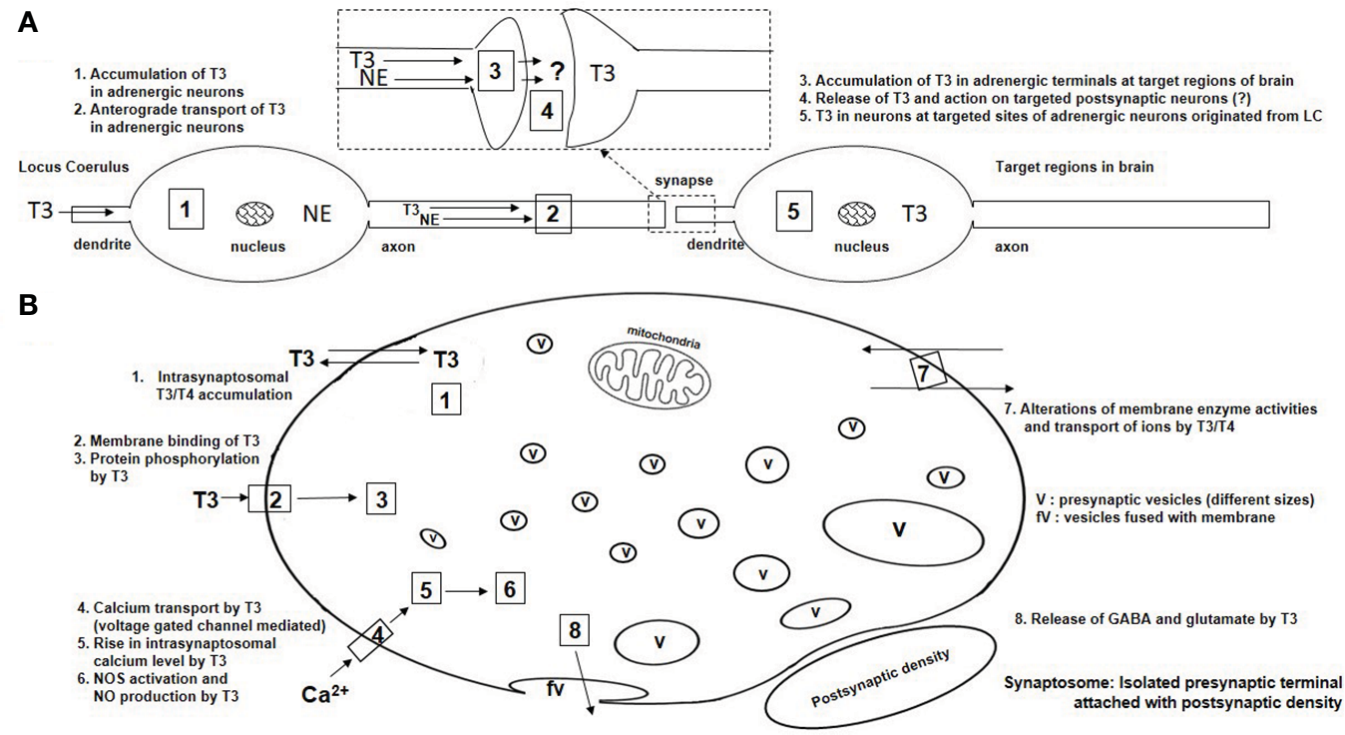
Schematic presentation of nongenomic actions of THs in neurons of adult mammalian brain. **(A)** Anterograde transport of T3 in adrenergic neurons at LC and accumulation of T3 in terminal target sites after T3 microinjection in the LC. The concept has been adopted from Dratman’s published research (**Figure 1**: level 4). **(B)** Synaptosomal actions of T3 in *in vivo* and *in vitro* experiments. The accumulation of T3 in synaptosomes (#1) was reported by Dratman (**Figure 1**, level 9). All facts (#1-8) are reported by other researchers (**Figure 1**, levels 10-19). v, vesicle; fv, fused vesicle.

Level 1-9 (**Figure 1**) demonstrates a summary of contributions of Dratman regarding development of the concepts of nongenomic action of TH in adult mammalian brain. These studies are summarized below (**Figure 2**). She provided insights into TH actions including (a) the adult brain responsiveness to TH, nullifying the earlier concept of non-responsiveness of adult brain to TH, (b) the maintenance of adult brain TH levels by deiodinase systems, and (c) iodothyronine production as catecholamine analogs and their possible action in brain, particularly in the case of thyroidal illness (16). Based on the experimental evidence since the 1970s (**Figure 1**), Dratman proposed the concept of nongenomic action of TH in adult brain [i.e., the sympathomimetic activity of “iodothyronine-derived neurohormones” in brain (17). Dratman identified, for the first time, the *in vivo* accumulation of radiolabeled T4/T3 at nerve terminals using synaptosomes isolated from adult rat brains after intravenous injections of T3/T4 (**Figure 2**) (6, 18–20). Additionally, her group reported that T4/T3 might act peripherally in the salivary gland (21) and centrally in autonomic nervous system for regulation of blood pressure (20) and heart rate (22).

Behavioral studies showed that TH might influence the circadian rhythm of temperature regulation (23) and have an impact on affective disorders (24) related to thyroidal dysfunctions.

Dratman extended her studies with findings of the *in vivo* localization of radiolabeled T4/T3 in different regions of adult rat brain (a) after injection of TH (6, 9, 19, 20, 25–31), (b) during altered thyroidal states (20, 32–37) and (c) after pharmacologically altered adrenergic systems in brain during euthyroid (6, 28, 34–36) and altered thyroid conditions (6, 34–37). The pharmacological interventions in their studies were induced by adrenergic uptake inhibitors such as desmethylimipramine (35–37) and reserpine (28), and the neurotoxic agent [N-(2-chloroethyl)-N-2-bromobenzylamine hydrochloride (DSP-4)] selective for adrenergic neurons in the locus coeruleus (6, 34). Notably, this series of reports supported the concept of accumulation of T3 in different regions of adult mammalian brain and its action as neurotransmitter and/or co-transmitter with adrenergic systems (**Figure 2**).

Dratman further examined brain homeostatic mechanisms to maintain the T3/T4 levels at nerve terminals under altered conditions (9, 33, 35, 37). Finally, she proposed the possibilities of postsynaptic action of THs or derivatives after their release from adrenergic nerve terminals (34, 38). In addition, her group reported that T3 can induce nongenomic action on neuronal activation of hippocampal cells, one of the target regions of adrenergic system in adult rat brain (14). Such findings help to explain the physiological or pathophysiological influences of TH in psychobehavioral control in adult mammalian brain.

#### 3.1.2 Works of other researchers

Separate *in vivo* and *in vitro* studies have been executed by other investigators using synaptosomes isolated from adult rodent brains, to find the exact mechanism of nongenomic action(s) of T3 at nerve terminals (see **Figure 1**: levels 10-19, **Figure 2**). These studies described several nongenomic actions exerted by TH, including protein phosphorylation, calcium-flux, NOS activity, putative membrane receptor binding, uptake and release of THs. Synaptosomes isolated from the whole brains of adult rats show deiodinase activities for conversion of T4 to rT3 and T3 to 3,3’T2 or 3’,5’T2 (39). T4 and T3 have been estimated in synaptosomes isolated from 11 different regions of adult rat brain (40). The adult rat brain shows thyroid homeostatic mechanisms at nerve terminals by increasing synaptosomal T4 and T3 levels (41, 42) during altered thyroidal conditions, particularly at initial stages of the altered conditions (43). Anti-depressant treatments alter T3 levels in synaptosomes isolated from frontal cortex (44), amygdala (45) and cortical areas (46) of adult rat brains. Therefore, these *in vivo* studies using isolated synaptosomes under different experimental conditions indicate that nerve terminals of adult mammalian brain have a capacity to maintain their T4/T3 levels for a certain extent of conditions.

In adult rat brain synaptosomal fractions, a Na^+^-dependent carrier-mediated uptake for T3 was demonstrated that involved both high-affinity and low-affinity transport systems. T4 was transported by a concentration-dependent but Na^+^-independent manner (47). One study showed that cortical synaptosomes can release T3, but not T4, under depolarized conditions through a Ca^2+^-dependent process, thus supporting the concept that T3 can act as a neurotransmitter (48). Still, additional research is needed to confirm this point. Furthermore, T3 enhances Ca^2+^-dependent release of GABA under depolarizing conditions (49). T3 enhances Na^+^-dependent tryptophan transport (50) and inhibits leucine (51, 52) or GABA (53) uptakes in synaptosomes. These *in vitro* studies show that T3 is transported at nerve terminals and/or alters the ion-dependent transport of amino acids and amino acid derivatives as rapid nongenomic actions of T3 at nerve terminals of adult mammalian brain.

T3 shows high-affinity binding to synaptosomal membranes (54–56) isolated from adult rat brain cerebral cortex. T3 inhibits Na^+^/K^+^-ATPase activity in cerebrocortical synaptosomal membranes (56) and thus can modulate the neuronal depolarization in adult rat brain (57, 58). T3 inhibits membrane bound ectonucleotidase in synaptosomes isolated from hippocampus of adult rat brain and modulates ATP hydrolyses (59). These studies indicate that T3 can act in a nongenomic fashion to minimize the ATP loss at the synaptic level. T3 stimulates the activities of Ca^2+^/Mg^2+^-ATPase (60) and acetylcholinesterase (61) in cerebrocortical synaptosomes. Hence, T3 may have a role in calcium homeostasis and the modulation of cholinergic neurotransmission in adult brain. T3 inhibits glutamate-induced Ca^2+^-uptake in synaptosomes isolated from mouse whole brain (62). Furthermore, T3 enhances depolarization-induced Ca^2+^-uptake (63) in synaptosomes isolated from rat cerebral cortex and causes a transient rise in intrasynaptosomal Ca^2+^-calcium levels (64) which indicates the nongenomic action of T3 on the Ca^2+^-dependent neurotransmission process. Altered thyroidal conditions also mobilize the synaptosomal Ca^2+^-level (65). Interestingly, the transient rise of intrasynaptosomal Ca^2+^-was found to be associated with synaptosomal nitric oxide synthase activation (64). This indicates that T3 can act through a nongenomic Ca^2+^-calcium-dependent nitric oxide (NO) signaling pathway at synaptic regions and thereby modulate neurotransmission. In addition, a series of *in vitro* experiments demonstrated the T3-induced Ca^2+^- and calmodulin-dependent synaptosomal protein phosphorylation (66–68) that underlies nongenomic cellular signaling pathway (**Figure 2**).

### 3.2 Implications of Dratman’s work on nongenomic action

Dratman’s work provided a variety of types of support for her idea that THs have nongenomic actions and have distinct signaling roles (6, 14, 18–20, 25, 34) in mature CNS.

#### 3.2.1 Implications of localization in nerve terminals

As mentioned in section 3.1.1, much of Dratman’s research investigated the anatomical and subcellular localization of radioactivity in adult rodent brain following injections of labeled THs (8,9,19, 20,25-31). Electron microscopic studies showed accumulation of radiolabeled THs in neuropil, especially in nerve terminal regions (9, 20, 28). In addition, subcellular fractionation by differential centrifugation of homogenates of brain showed that the radiolabeled THs were concentrated in synaptosomes (6, 18–20).

The subcellular localization of THs points to the potential role of the compounds at the synapse. Dratman therefore espoused the hypothesis that THs (or their derivatives) might have neurotransmitter-like actions. The localization in nerve terminals readies the compounds for release into the synaptic cleft, where they might have influences on postsynaptic or presynaptic receptors. Thus, the THs are optimally positioned to participate in a synaptic signaling role.

#### 3.2.2 Implications of axonal transport

Thaw-mount autoradiography indicated that following intravenous (IV) administration of ^125^I-T3, radioactivity corresponding to T3 (80%) or other iodinated organic compounds (15%) accumulated in discrete brain regions. At 10 hours post-injection, the radiolabel shifted to fiber tracts, implying that it is transported along axons (25). Additional studies showed that IP administration of the DSP-4, toxin specific to adrenergic neurons in locus coeruleus (LC), reduced the distribution of T3 immunohistochemistry in specific sites in the forebrain, the target site of the adrenergic neurons originated from LC (34). These data suggest that DSP-4 disrupted the transport of T3 throughout the brain and indicate that the transport is orthograde. Since orthograde axonal transport requires energy (69), such transport of T3 may indicate the importance of the hormone for actions at the nerve terminal, in keeping with a signaling function at the synapse.

#### 3.2.3 Implications of localizations of THs in brain areas

The concentration of radioactivity in discrete brain regions following administration of radiolabeled THs suggests that the actions of the hormones or their derivatives are specific to particular brain functions under particular circumstances in adult mammalian brain. These actions could be either nongenomic or genomic.

##### 3.2.3.1 Implications of locus coeruleus in actions of TH

As mentioned in 3.2.2, injections of LC-specific toxin DSP-4 depleted the T3 immunoreactivity in the LC and in noradrenergic projection sites throughout the brain (34). LC cell bodies are thought to be noradrenergic, since a lesion of the LC depletes most of the NE throughout the contralateral forebrain (70). As a result of these findings, Dratman hypothesized that T3 in brain is a co-transmitter with NE (34).

After the discovery of iodothyronamines in brain (71), Dratman and her colleagues made a comprehensive study of 3-T1AM in the LC (38). Microinfusion of 3-T1AM into the LC dose-dependently increased neuronal firing rates in 62% of the responsive neurons. IV injection of radiolabeled 3-T1AM resulted in radiolabeling over discreet brain areas, including LC, cortical areas and mammillary bodies. Since 3-T1AM has actions which generally oppose the effects of THs, it may be concluded that the 3-T1AM-induced increases in cell firing in the LC have negative feedback activities to regulate output of the LC.

##### 3.2.3.2 Implications of the role of the hippocampus in TH actions

The series of *in vivo* studies by Dratman et al. indicates that T3 is strongly accumulated in the hippocampal formation (6, 9, 20, 25, 26, 28–31, 34), particularly in pyramidal cells (26) at the cornu ammonis (CA1 and CA3) and in the granular layer of the dentate gyrus (20, 25) with pericellular neuropil in dentate gyrus (20). As described above (section 3.2.3.1), the experimental evidence accumulated by Dratman et al. indicates that TH interacts with adrenergic neurons originating from the LC and modulates adrenergic neurotransmission at their target sites. The LC provides noradrenergic neuronal connections to hippocampal glutamatergic pyramidal cells, to GABAergic interneurons in the CA1-CA3 connections and to glutamatergic granular cells in the dentate gyrus of the hippocampus (72).

Dratman et al. (14) conducted electrophysiological experiments with adult rats using microelectrodes inserted in (a) the dentate gyrus *in vivo* and (b) in CA1 of hippocampal slices isolated from adult rat brain during *in vitro* experiments. The electrophysiological recordings were analyzed to find the changes in (a) population spikes and excitatory postsynaptic potentials (EPSPs) *in vivo* and (b) cellular firings generated by the pyramidal cell layer *in vitro.* Euthyroid and hypothyroid animals were used for both experimental conditions. The effects of T4 microinjections were analyzed with the *in vivo* experiments. The effects of T4 and T3 microinjections on prior and post applications of NE were investigated during the *in vitro* experiments. These experimental findings showed that T4 and T3 had opposite effects, as described below.

(a) T4 inhibited field potentials *in vivo*, with pronounced effects in the hypothyroid condition and suppressed the stimulatory response of NE *in vitro* on the cellular firings.

(b) T3 enhanced the stimulatory response of NE *in vitro* on the cellular firings.

(c) T4, T3 and NE were without effect *in vitro* on the cellular firings during hypothyroid preparations.

Based on these results, Dratman et al. (14) concluded that the cellular forms of THs might be the key factors for rapid nongenomic action of TH on the neuronal excitability in the hippocampus. Such a nongenomic action of TH might involve the adrenergic system. The adrenergic system appeared to be very low or absent in hypothyroidism due to a reduction of adrenergic activity during the hypothyroid condition.

The hippocampus has a widely accepted role in the mechanisms of learning and memory (73). The effects of hypothyroidism include an inhibitory influence on learning and memory (74–76). These findings imply that the hippocampus is an important target tissue for effects of THs on learning and memory. The almost instantaneous electrophysiological effects of THs strongly support a nongenomic mechanism.

## 4 Conclusions

The present review of literature analyses the putative mode of nongenomic action of TH at nerve terminals of adult mammalian brain. Roughly half of the studies in this area belong to Dratman et al. TH appears to function in multiple forms including T3 itself and other derivatives like rT3 and 3-T1AM depending on the conditions. The experimental evidence supports the idea that T3 can act as neurotransmitter and modulate uptake/release of other neurotransmitters and ions at synaptic regions. T3 can elicit the nongenomic signalling including calcium-dependent and NO-mediated pathways in synapses. T3 may act through membrane binding and/or other non-nuclear receptors, which remain unidentified. Notably, it is a burning issue whether TH acts presynaptically and/or postsynaptically at nerve terminals and how it is associated with long-term potentiation related to memory formation and cognitive functions. The exact pathophysiological mechanisms of TH at nerve terminals in adult brain would unveil a new horizon of neuroscience research concerning better treatment strategies for cognitive dysfunctions related to dysthyroidism.

## Author contributions

All authors contributed to the conception of the manuscript. All authors contributed to the drafting and revising of the text. All authors approve of the publication of the manuscript and accept responsibility for the accuracy of the statements therein. All authors contributed to the article and approved the submitted version.
